# CXCR4 can induce PI3Kδ inhibitor resistance in ABC DLBCL

**DOI:** 10.1038/s41408-018-0056-9

**Published:** 2018-02-22

**Authors:** Joo Hyun Kim, Won Seog Kim, Kyung Ju Ryu, Seok Jin Kim, Chaehwa Park

**Affiliations:** 1 Department of Health Sciences and Technology, Samsung Advanced Institute for Health Sciences and Technology, Sungkyunkwan University, Seoul, 06351 Korea; 2 Division of Hematology and Oncology, Department of Medicine, Samsung Medical Center, Sungkyunkwan University School of Medicine, Seoul, 06351 Korea; 3 Research Institute for Future Medicine, Samsung Medical Center, Sungkyunkwan University School of Medicine, Seoul, 06351 Korea

Diffuse large B-cell lymphoma (DLBCL) is the most common type of non-Hodgkin lymphoma. DLBCL represents a highly heterogeneous group of tumors with variable clinical features, molecular genetic alterations, therapeutic responsiveness levels, and prognosis^1^. The pathway involving the key components phosphatidyl inositol-3-kinase (PI3K), protein kinase B (PKB/AKT) and mammalian target of rapamycin (mTOR) plays a major role in regulation of proliferation and cell death of DLBCL^2,3^. PI3Ks play multiple roles, ranging from cell growth and proliferation to migration. Dysregulation of the PI3K pathway is associated with numerous cancers^4,5^. Here, we have focused on the contribution of the *δ* isoform of PI3K, which plays a central role in normal B-cell development and function. PI3Kδ signaling is overactive in many B-cell malignancies and shown to drive proliferation, survival, and trafficking to lymphoid tissue. The most effective single PI3K-based therapeutic agent is idelalisib, a p110δ-selective inhibitor. Idelalisib has achieved notable success in clinical trials for patients with relapsed chronic lymphocytic leukemia or indolent lymphoma^6,7^. However, treatment with idelalisib alone is insufficient due to induced resistance, clearly highlighting the urgent need to identify effective new targets for combination therapy.

Stromal-derived factor (SDF-1 or CXCL12), a CXC chemokine, and its cognate seven transmembrane G-protein coupled receptor 4 (CXCR4) play significant roles in promoting migration and trafficking of malignant B cells in hematological malignancies. CXCR4 has been detected in several tissues, in particular hematopoietic cells^8,9^. Additionally, CXCR4 expression is a marker of DLBCL recurrence and associated with shorter survival^10^. Here, we established idelalisib-resistant ABC-DLBCL cells and addressed the functional consequences of CXCR4 expression.

To determine the molecular targets in primary resistance to idelalisib, we isolated surviving cells after exposing three DLBCL cell lines (Supplementary Material 1) to lethal doses of idelalisib (>20 μM) for 3 days. The primary refractory ABC-DLBCL cell lines, Riva-Idela(pR), U2932-Idela(pR) and OCI-Ly10-Idela(pR), were more resistant to idelalisib (Fig. 1a) than parental cells (Supplementary Material 2). Genome-wide analysis of gene transcripts expressed in the three pairs of primary refractory and respective parental cell lines was performed using Illumina HumanHT-12 v4 Expression BeadChip (Fig. 1b, Supplementary Material 3). Notably, expression levels of 24 genes were augmented more than two-fold in the three sets (Supplementary Table 1). Among these, we selected the CXCR4 gene that plays important roles in hematological tumor cell survival, migration and interactions with the protective microenvironment. Increased expression of CXCR4 in idelalisib-treated surviving cells was confirmed using Western blot (Fig. 1c, Supplementary Material 4) and FACS analyses (Fig. 1c, Supplementary Material 5) in all three B-cell lymphoma cell lines. Primary refractory groups (Riva-Idela(pR), U2932-Idela(pR) and OCI-Ly10-Idela(pR)) also showed significantly increased migration capacity, compared to control groups, in the presence of CXCL12 (Supplementary Figure 1, Supplementary Material 6). Our results clearly demonstrate that CXCR4 expression is correlated with primary refractory idelalisib resistance of DLBCL cells. U2932 and OCI-Ly10 cells express lower levels of CXCR4 than Riva, however, they are extremely resistant than Riva cells (Fig. 1c). Therefore the levels of CXCR4 expression and idelalisib resistance are not correlated among lymphoma cell lines harboring different genetic backgrounds. To gain insights into the molecular mechanisms underlying the involvement of CXCR4 in idelalisib resistance of ABC DLBCL cell lines, we analyzed the expression of signaling-related genes. Notably, increased p-AKT and p-MAPK expression was evident in idelalisib-resistant cells (Fig. 1d). To ascertain whether CXCR4 promotes AKT and MAPK activation, primary refractory cells were incubated with a CXCR4-neutralizing antibody or the CXCR4 inhibitor, AMD3100. Western blot revealed blockage of AKT and MAPK activation processes upon inhibition of CXCR4 (Figs. 1e, f). The data collectively suggest that CXCR4 expression in idelalisib-resistant cells contributes to AKT and MAPK activation. To further determine the biological significance of the above findings, we examined the viability of DLBCL cells in the presence of idelalisib combined with the CXCR4 inhibitor, AMD3100. Co-administration of idelalisib and AMD3100 led to a significant decrease in viability of the primary refractory cell lines (Riva-Idela(pR), U2932-Idela(pR) and OCI-Ly10-Idela(pR)) that display CXCR4 overexpression (Fig. 2a, Supplementary Figure 2a, Supplementary Material 7). Consistently, co-treatment with idelalisib and AMD3100 induced apoptosis, as evident from the increased number of Annexin V-positive cells and cleavage of caspase 3/7 in primary refractory cell lines (Supplementary Figure 2b, Supplementary Material 2). Additionally, isolated primary refractory cells from samples derived from two DLBCL patients (Patients #1 and #2) showed increased expression of CXCR4, p-AKT, p-p70S6K, p-MAPK, BCL-xL, and CD79B (Fig. 2b). Consistent with this finding, treatment with a combination of idelalisib and CXCR4 inhibitor (AMD3100) or everolimus for 48 h was more efficacious for patient-derived primary refractory DLBCL (pR) (Figs. 2c, d, Supplementary Figure 3). Combined treatment with idelalisib and everolimus or AMD3100 resulted in a synergistic reduction in cell viability with CI < 1.0. Supplementary Figures 2 and 3 shows a Fraction-affected-Combination index plot created using CompuSyn software.

**Fig. 1:**
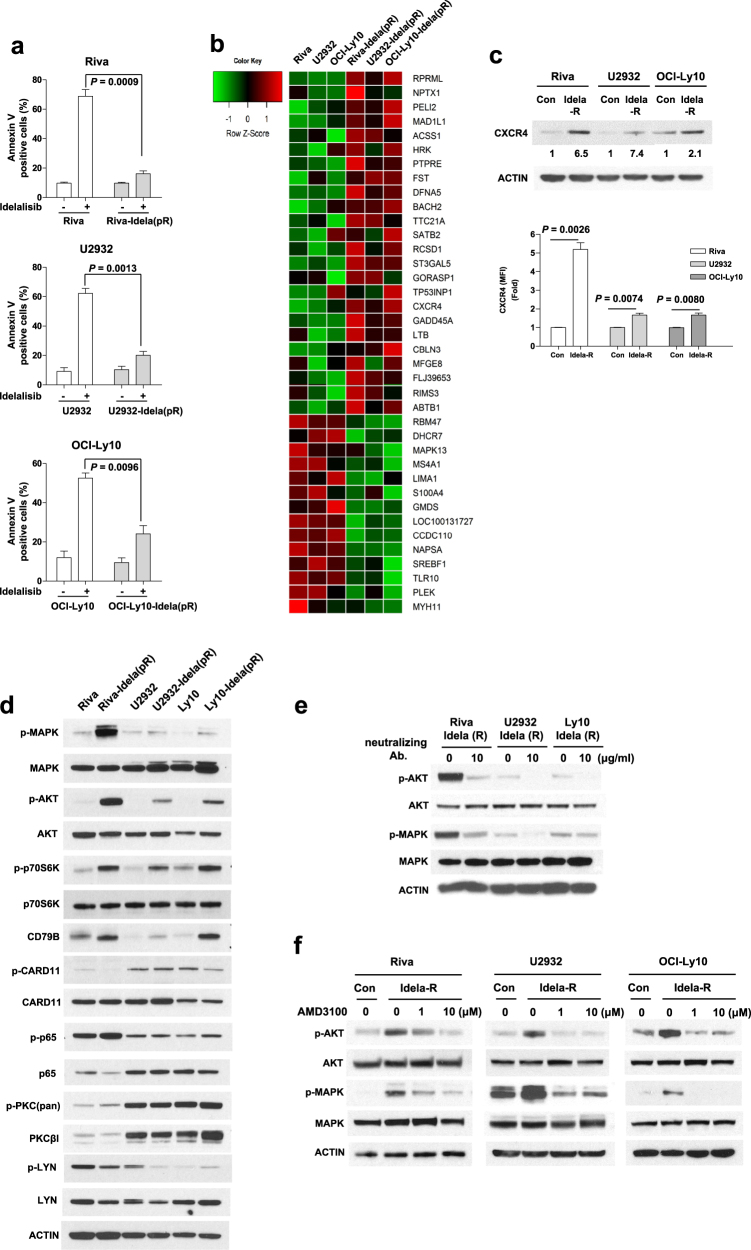
CXCR4 expression is upregulated and associated with differential activation of AKT, MAPK and p70S6K in primary refractory cells, Riva-Idela(pR), U2932-Idela(pR) and OCI-Ly10-Idela(pR). **a** Cells were treated with idelalisib (10 μM) for 48 h, and the percentage of apoptotic cells monitored with annexinV/propidium iodide staining. **b** Gene expression analysis of primary refractory (Riva-Idela(pR), U2932-Idela(pR) and OCI-Ly10-Idela(pR)) and parental control cells (Riva, U2932 and OCI-Ly10). **c** Western blot and FACS analysis of CXCR4 in primary refractory and parental controls. Western blots were quantified via densitometry and the results are presented as fold change. **d** Activation of signaling proteins, including AKT, MAPK, and p70S6K, from datasets observed with Western blot. **e**, **f** Western blot analysis of p-AKT and p-MAPK-following treatment with CXCR4-neutralizing Ab. (10 μg/mL; 24 h) and AMD3100 (1, 10 μM; 4 h) in primary refractory cells

**Fig. 2:**
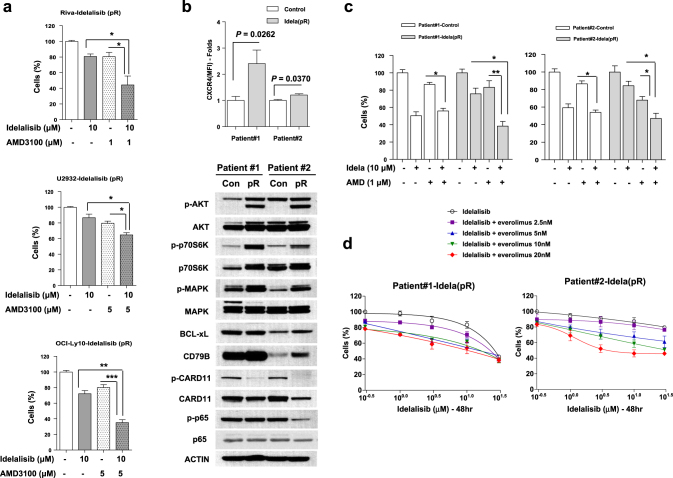
Combined inhibition with AMD3100 and idelalisib leads to synergistic effects in circumventing primary refractory resistance of cell lines and patient-derived primary human ABC-DLBCL. **a** Primary refractory ABC-DLBCL cell lines were treated with idelalisib (10 μM) in the presence or absence of AMD3100 (Riva -Idela(pR), 1 μM; U2932 and OCI-Ly10-Idela(pR), 5 μM) for 48 h. Cell viability was evaluated via trypan blue staining. **b** FACS analysis of CXCR4 expression and Western blot analysis of p-AKT, p-p70S6K, p-MAPK, p-p65, p-CARD11, BCL-xL, and CD79B in primary human DLBCL cells and primary refractory subpopulations (pR) isolated as described in the Methods section. **c** Two of primary human DLBCL(pR) cells were resuspended in medium containing 10% FBS and treated with idelalisib (10 μM) in the presence or absence of AMD3100 (1 μM) for 48 h. Viability was monitored based on trypan blue staining and the percentage of live cells normalized to untreated controls. (P#1, Patient #1; P#2, Patient #2). Patient-control means patient samples that are naïve to idelalisib. Data are presented as means ± standard deviation of triplicate values. Probability values of the *t*-test are presented (*P < 0.05; **P < 0.01; ***P < 0.001). **d** Single agent and combination responses evaluated with the CCK-8 assay. Combination responses evaluated with the CCK-8 assay and isobologram analysis are presented in Supplementary Figure 3

Previous reports have shown that stromal cells in the bone marrow microenvironment support CLL survival and protect cells from chemotherapy-induced apoptosis^11,12^. To determine the interactions between ABC DLBCL cells and the bone marrow microenvironment in vitro and their effects on idelalisib sensitivity, ABC DLBCL cell lines were treated with idelalisib in the presence of primary bone marrow-derived stromal cells (BMSCs) (Supplementary Material 8). CXCR4 expression was consistently increased in the presence of BMSCs (Supplementary Figure 4a). The combination of AMD3100 and idelalisib profoundly inhibited primary refractory DLBCL cell growth, even in the presence of BMSCs (Supplementary Figure 4b). In contrast, the combined effect of AMD3100 and idelalisib was not evident on parental cells in the presence or absence of BMSCs (Supplemental Figure 5). To investigate primary refractory mechanisms for other PI3K inhibitors, we collected surviving cells after treatment with buparlisib (pan-PI3K inhibitor) or copanlisib (PI3K α and δ inhibitor). As shown in Supplementary Figure 6a, buparlisib and copanlisib-resistant primary refractory Riva and U2932 cells showed increased cell surface expression of CXCR4. Furthermore, combined effects of AMD3100 and buparlisib or copanlisib were observed in resistant cells (Supplementary Figure 6b). These results support the potential utility of CXCR4 as a promising target for different types of PI3K inhibitor- resistant DLBCL.

To clarify the changes in the signaling pathway induced by long-term treatment with idelalisib, we established acquired idelalisib-resistant DLBCL cell lines. Cell lines with acquired idelalisib resistance (termed Riva-Idela(0.3 μM), U2932-Idela(3 μM), and OCI-Ly10-Idela(3 μM)) were generated by exposing each parental cell line to progressively increasing concentrations of idelalisib for 4 weeks. The cell lines established were more resistant to idelalisib and displayed superior ability to form colonies (Supplementary Figure 7a & 7b) relative to parental cells (Supplementary Material 9). Next, we investigated whether the enhanced resistance effect is associated with activation of specific signaling pathways. Notably, cells with acquired resistance showed upregulation of p-CARD11, p-p65, p-MAPK, BCL-xL, and p-p70S6K (Supplementary Figure 7c). Microarray analysis revealed that 26 NF-κB pathway-related genes were increased in Riva and U2932 acquired idelalisib-resistant cells (Supplementary Table 2), while six mTOR pathway-related genes increased in OCI-Ly10 acquired idelalisib-resistant cells (Supplementary Table 3). The established idelalisib-resistant cells exhibited 2 to 14-fold higher NF-κB transcriptional activity than their respective parental cell counterparts (Supplementary Figure 7d, Supplementary Material 10). Activation of other NF-κB family proteins, including IκB and IKK, was not correlated with NF-κB activation (Supplementary Figure 8). Based on these results, we hypothesize that acquired idelalisib resistance is associated with the NF-κB and mTOR pathways. Accordingly, we further examined whether velcade, an NF-κB inhibitor, and everolimus, an mTOR inhibitor, exert synergistic effects with idelalisib. As shown in Supplementary Figure 9a and 9b, combination of either velcade or everolimus with idelalisib induced synergistic growth inhibition in Riva and U2932 acquired resistant cells, while everolimus plus idelalisib was effective for p-p70S6K-high OCI-Ly10 resistant cells.

In the current study, we identified CXCR4 as a cause of primary resistance to idelalisib in ABC DLBCL via gene expression analysis, consistent with earlier studies showing that CXCR4 is associated with drug resistance of cancer cells^13,14^. Primary refractory cells expressed high levels of CXCR4 on the cell surface and application of CXCR4 antagonists combined with idelalisib resulted in a profound synergistic effect of growth inhibition, compared to individual treatment. However, in acquired resistant cell lines, CXCR4 expression was gradually decreased during long-term idelalisib treatment and selection of resistant cells (Supplementary Figure 10). The NF-κB and mTOR pathways were activated in acquired resistant cells and NF-κB or mTOR inhibitors successfully sensitized resistant cells to idelalisib. In view of these findings, we speculate that activation of alternative signaling pathways may be responsible for acquired resistance. Therefore, p65 activation upon long-term idelalisib treatment of Riva and U2932 cells may explain the greater response of these cells to velcade. Moreover, the significant inhibitory effect of everolimus combined with idelalisib achieved in OCI-Ly10 resistant cell lines could be explained by increased expression of the mTOR target, p70S6K. Recently, different CXCR4 inhibitors have been successfully combined with idelalisib in non-Hodgkin lymphoma cell lines to reduce growth and suppress tumor progression^10^. Idelalisib resistance in Waldenstrom’s Macroglobulinemia cells has been overcome through BCL-2 targeting^15^. However, in our experiments, BCL-2 expression was not increased (Supplementary Figure 7c) and inhibition of BCL-2 with ABT-199 did not affect survival of idelalisib-resistant ABC DLBCL (data not shown). Our results provide new insights into the mechanisms underlying primary as well as acquired idelalisib resistance in ABC-DLBCL. The signaling pathways activated vary according to type of resistance (primary and secondary) and cell characteristic. To prevent primary resistance or overcome secondary resistance to PI3K inhibitors, a strategy involving combined targeting of alternative activated pathways may present a superior therapeutic approach for ABC-DLBCL.

## Electronic supplementary material

Supplementary tables and figures

Supplementary materials and method
