# Supplementary material for: CXCR4 can induce PI3Kδ inhibitor resistance in ABC DLBCL
Source: Blood Cancer J. 2018 Feb 22;8(2):23. doi: 10.1038/s41408-018-0056-9 (PMC5823878; doi:10.1038/s41408-018-0056-9)
Supplement: Supplementary file 1 — Supplementary tables and figures [file 41408_2018_56_MOESM1_ESM.docx]

**SUPPLEMENTARY TABLES AND FIGURES**

**CXCR4 can induce PI3Kδ inhibitor resistance in ABC DLBCL**

Joo Hyun Kim^1^, Won Seog Kim^1,2,*^, Kyungju Ryu1, Seok Jin Kim^1,2^ and Chaehwa Park^3,*^

^1^Department of Health Sciences and Technology, Samsung Advanced Institute for Health Sciences and Technology, Sungkyunkwan University, Seoul 06351, Korea

^2^Division of Hematology and Oncology, Department of Medicine, Samsung Medical Center, Sungkyunkwan University School of Medicine, Seoul 06351, Korea

^3^ Research Institute for Future Medicine, Samsung Medical Center, Sungkyunkwan University School of Medicine, Seoul 06351, Korea

*Correspondence: Won Seog Kim and Chaehwa Park, Division of Hematology and Oncology, Department of Medicine, Samsung Medical Center, Sungkyunkwan University School of Medicine, 50 Irwon-dong, Seoul 06351, Korea; e-mail: [wskimsmc@skku.edu](mailto:wskimsmc@skku.edu) (Tel: +82 2 3410 6548; fax: +82 2 3410 1757) and [cpark@skku.edu](mailto:cpark@skku.edu) (Tel: +82 2 3410 3458; fax: +82 2 3410 0041)

**Supplementary Table 1.** Upregulation of related genes in primary refractory Riva, U2932 and OCI-Ly10 cell lines.

**Supplementary Table 2.** NF-κB pathway-related genes in acquired idelalisib-resistant Riva and U2932 cell lines.

**Supplementary Table 3.** mTOR pathway-related genes in OCI-Ly10-acquired idelalisib-resistant cell lines.

**Supplementary Figure 1.** Migrating cells were counted via FACS after 4 h. Fold change of migrating cells relative to the number of parental control cells. Data are presented as means ± standard deviation of triplicate values. Probability values of the t-test are presented ( *P* < 0.05 ).

**Supplementary Figure 2.** Activity of idelalisib in combination with AMD3100 (a) Single agent and combination responses evaluated with the CCK-8 assay and isobologram analysis. IC_50_ value is the concentration obtained when idelalisib was treated for 72 hr. Matrix block experiment to determine viability, estimated with the CCK-8 assay. Analysis of interactions of AMD3100 and idelalisib using CalcuSyn software (Biosoft, Ferguson, MO, USA). Combination index defining the interactions between AMD3100 and idelalisib are plotted against the fraction of growth-inhibited cells. CI values >1 signify antagonism, =1 additivity, and <1 synergy. (b) Apoptosis was detected using annexin V/propidium iodide staining. Caspase-3/7 enzymatic activity was measured using a luminometer.

**Supplementary Figure 3.** Combination of everolimus and idelalisib exerts synergistic inhibitory effects in primary refractory resistant patient sample cells Single agent and combination responses evaluated with the CCK-8 assay and isobologram analysis. Matrix block experiment to determine viability, estimated with the CCK-8 assay. Analysis of interactions of everolimus and idelalisib using CalcuSyn software (Biosoft, Ferguson, MO, USA). Combination index defining the interactions between everolimus and idelalisib are plotted against the fraction of growth-inhibited cells. CI values >1 signify antagonism, =1 additivity, and <1 synergy.

**Supplementary Figure 4.** Combination of AMD3100 and idelalisib promotes ell death in the presence of BMSCs. Primary refractory, Riva-Idela(pR), U2932-Idela(pR) and OCI-Ly10-Idela(pR) cells were co-cultured in serum-reduced conditions (1% FBS) in the absence or presence of BMSCs. Viable CD20+ DLBCL cells were determined via FACS using PI exclusion. (a) FACS analysis of CXCR4 surface expression in resistant cells. (b) Primary refractory Riva-Idela(pR), U2932-Idela(pR) and OCI-Ly10-Idela(pR) cells were cultured with stromal cells in the presence or absence of AMD3100, idelalisib or both reagents for 48 h. Data are presented as means ± standard deviation of triplicate values. Probability values of the t-test are presented (*, *P*< 0.05; **, *P* < 0.01; ***, *P* < 0.001; n.s., *P* > 0.05).

**Supplementary Figure 5.** Combination of AMD3100 and idelalisib promote cell death in the presence of BMSCs. Parental Riva, U2932 and OCI-Ly10 cells were co-cultured in serum-reduced conditions (1% FBS) in the absence or presence of BMSCs. Viable CD20+ DLBCL cells were determined via FACS using PI exclusion. Cells were cultured with stromal cells in the presence or absence of AMD3100, idelalisib or both reagents for 48 h. Data are presented as means ± standard deviation of triplicate values. Probability values of the t-test are presented (*, *P*< 0.05; n.s., *P* > 0.05).

**Supplementary Figure 6.** Combination of AMD3100 and idelalisib exerts synergistic inhibitory effects in primary refractory resistant cells against pan-PI3K and PI3K α and δ inhibitor. Parental and primary refractory Riva and U2932 cell lines were treated with buparlisib (300nM) and copanlisib (Riva(pR), 3nM; U2932(pR), 30nM) in the presence or absence of AMD3100 (Riva(pR), 1μM; U2932(pR), 5μM) for 48 h. (a) FACS analysis of surface CXCR4 expression in primary refractory cells compared to parental controls. (b) Cell viability was evaluated via trypan blue staining. Data are presented as means ± standard

deviation of triplicate values. Probability values of the t-test are presented (*, *P*< 0.05; **, *P* < 0.01; n.s., *P* > 0.05).

**Supplementary Figure 7.** Long-term exposure to idelalisib activates NF-κB and mTOR contributing to acquired resistance. (a) Dose-response curve of ABC-DLBCL cell lines to idelalisib. Cells were treated with the indicated doses of idelalisib for 48 h, and subjected to the CCK-8 assay. (b) Colony formation of parental and acquired cells. (c) Western blot to monitor the expression of BCR signaling-related proteins in parental ABC-DLBCL and corresponding cells with acquired resistance. (d) Parental or resistant cells were transfected with a NF-κB driven luciferase reporter. After incubation for 24 h, cells were subjected to the luciferase assay. Data are presented as means ± standard deviation of triplicate values. Probability values of the t-test are presented (*P* < 0.05 ).

**Supplementary Figure 8.** Western blot to monitor expression levels of NF-κB, IκB, and IKK family proteins in parental ABC-DLBCL and corresponding cells with acquired resistance.

**Supplementary Figure 9.** Activity of idelalisib in combination with velcade (a) or everolimus (b). Single agent and combination responses evaluated with the CCK-8 assay and isobologram analysis. Matrix block experiment to determine viability, estimated with the CCK-8 assay. Analysis of interactions of velcade or everolimus and idelalisib using CalcuSyn software (Biosoft, Ferguson, MO, USA). Combination index defining the interactions between velcade or everolimus and idelalisib are plotted against the fraction of growth-inhibited cells. CI values >1 signify antagonism, =1 additivity, and <1 synergy. Each *P* value of the growth inhibition graph showed significance, compared to single treatment with idelalisib (*P* < 0.05).

**Supplementary Figure 10.** CXCR4 expression is gradually decreased with exposure time. Surface CXCR4 expression was determined via FACS analysis. Primary refractory cells were cultured in the presence of idelalisib (Riva, 0.3μM; U2932 & OCI-Ly10, 3μM) for 4 weeks. Probability values of the t-test are presented, compared to 0 day (**P*< 0.05; n.s., *P*> 0.05).

**Supplementary Table 1.**

| **Gene**  **Symbol** | **Fold change** | | | **Gene**  **Symbol** | **Fold change** | | |
| --- | --- | --- | --- | --- | --- | --- | --- |
|  | **Riva-Idela(pR)** | **U2932-**  **Idela(pR)** | **OCI-Ly10-**  **Idela(pR)** |  | **Riva-**  **Idela(pR)** | **U2932-**  **Idela(pR)** | **OCI-Ly10-**  **Idela(pR)** |
| **RPRML** | 8.8 | 4.4 | 15.8 | **RCSD1** | 4.0 | 2.5 | 2.9 |
| **NPTX1** | 15.6 | 2.9 | 2.2 | **ST3GAL5** | 4.1 | 2.1 | 3.1 |
| **PELI2** | 10.3 | 2.4 | 4.9 | **GORASP1** | 2.7 | 2.2 | 4.1 |
| **MAD1L1** | 5.9 | 3.0 | 5.1 | **TP53INPI** | 4.7 | 2.2 | 2.0 |
| **ACSS1** | 5.2 | 2.1 | 6.4 | **CXCR4** | 4.2 | 2.1 | 2.4 |
| **HRK** | 6.2 | 4.0 | 2.1 | **GADD45A** | 3.7 | 2.2 | 2.6 |
| **PTPRE** | 5.7 | 2.2 | 4.0 | **LTB** | 2.4 | 3.5 | 2.6 |
| **FST** | 4.9 | 2.0 | 4.9 | **CBLN3** | 2.4 | 2.7 | 3.1 |
| **DFNA5** | 5.6 | 2.1 | 3.5 | **MFGE8** | 3.2 | 2.3 | 2.5 |
| **BACH2** | 5.7 | 3.1 | 2.0 | **FLJ39653** | 2.6 | 2.5 | 2.8 |
| **TTC21A** | 4.5 | 2.3 | 3.8 | **RIMS3** | 2.2 | 2.5 | 3.0 |
| **SATB2** | 4.5 | 3.1 | 2.2 | **ABTB1** | 3.4 | 2.1 | 2.1 |

**Supplementary Table 2.**

| **Gene**  **Symbol** | **Fold change** | | **Gene**  **Symbol** | **Fold change** | |
| --- | --- | --- | --- | --- | --- |
|  | **Riva-Idela(0.3μM)** | **U2932-Idela(3μM)** |  | **Riva-Idela(0.3μM)** | **U2932-Idela(3μM)** |
| **PLAC8** | 3.2 | 2.0 | **FAM69B** | 2.0 | 3.9 |
| **ITGB2** | 2.2 | 2.2 | **PELI2** | 2.5 | 2.1 |
| **SERPINE2** | 2.9 | 2.4 | **CLIP3** | 2.3 | 2.5 |
| **TRPC1** | 2.0 | 2.5 | **HCP5** | 2.7 | 3.7 |
| **RPRML** | 3.5 | 9.9 | **BAIAP3** | 2.2 | 2.7 |
| **ADARB1** | 6.8 | 2.1 | **MIAT** | 2.8 | 2.7 |
| **TXNIP** | 3.2 | 3.6 | **GADD45A** | 2.3 | 2.0 |
| **ST3GAL5** | 2.4 | 2.1 | **BACH2** | 2.4 | 2.2 |
| **TCEA3** | 2.3 | 2.9 | **HRK** | 2.3 | 3.4 |
| **TSPAN9** | 3.9 | 2.8 | **FGR** | 2.3 | 2.0 |
| **DLL3** | 3.4 | 2.3 | **RNU1-5** | 2.6 | 7.2 |
| **RN7SK** | 3.7 | 2.5 | **RNU1G2** | 2.9 | 7.7 |
| **TSC22D3** | 3.0 | 2.2 | **RNU11** | 2.9 | 3.0 |
| **ALDOC** | 3.1 | 2.8 | **RNU1-3** | 2.8 | 7.5 |

**Supplementary Table 3.**

| **Gene**  **Symbol** | **Fold change** | **Gene**  **Symbol** | **Fold change** |
| --- | --- | --- | --- |
|  | **OCI-Ly10- Idela(3μM)** |  | **OCI-Ly10- Idela(3μM)** |
| **PTEN** | 11.5 | **TNF** | 3 |
| **RPS6KA2** | 8.3 | **PRKCB** | 3.4 |
| **RRAGD** | 7 | **PRKCB1** | 3 |

**Supplementary Figure 1.**

**
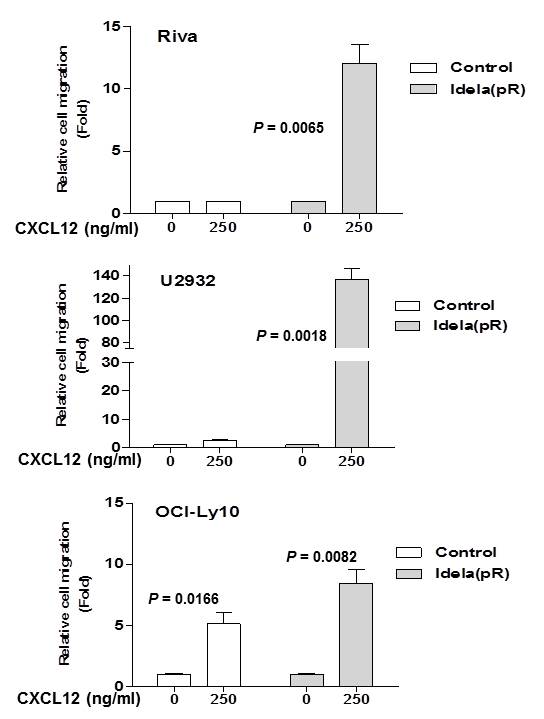
**

**Supplementary Figure 2.**

**
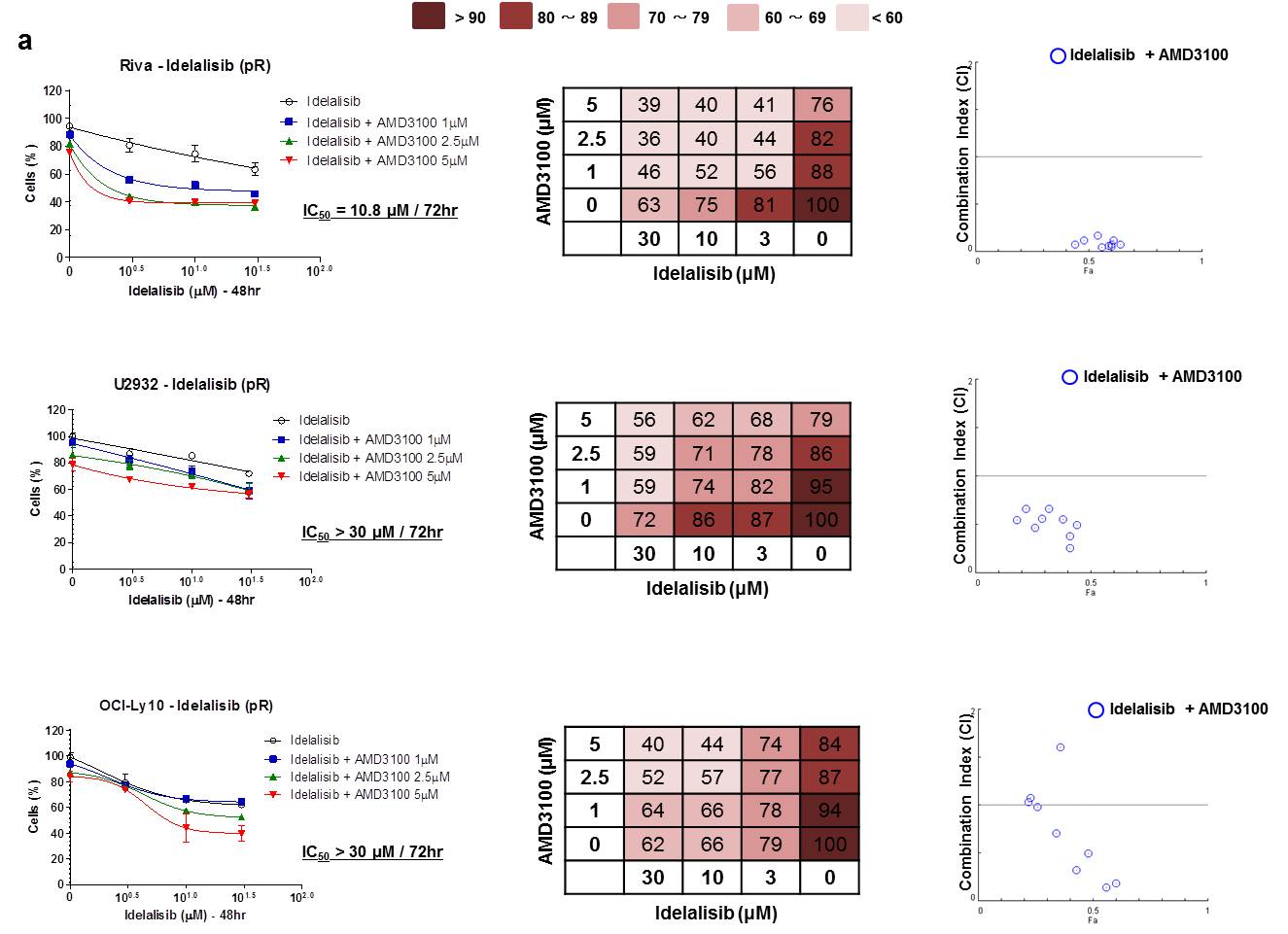
**

**
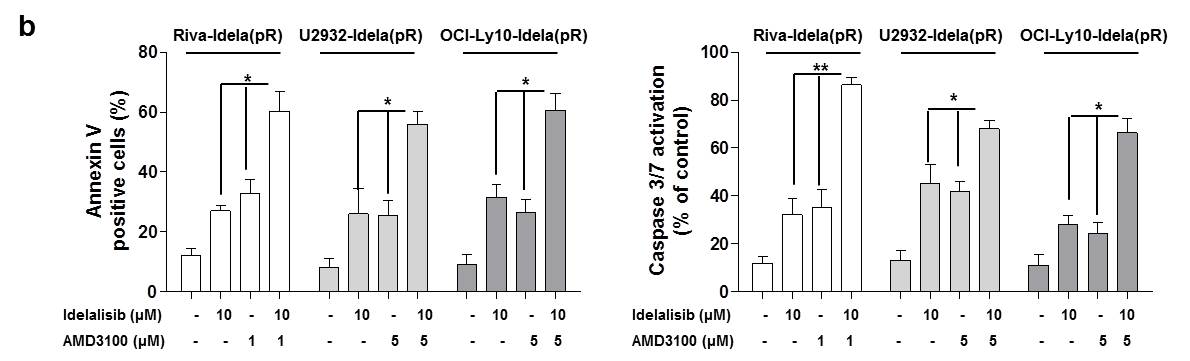
**

**Supplementary Figure 3.**

**
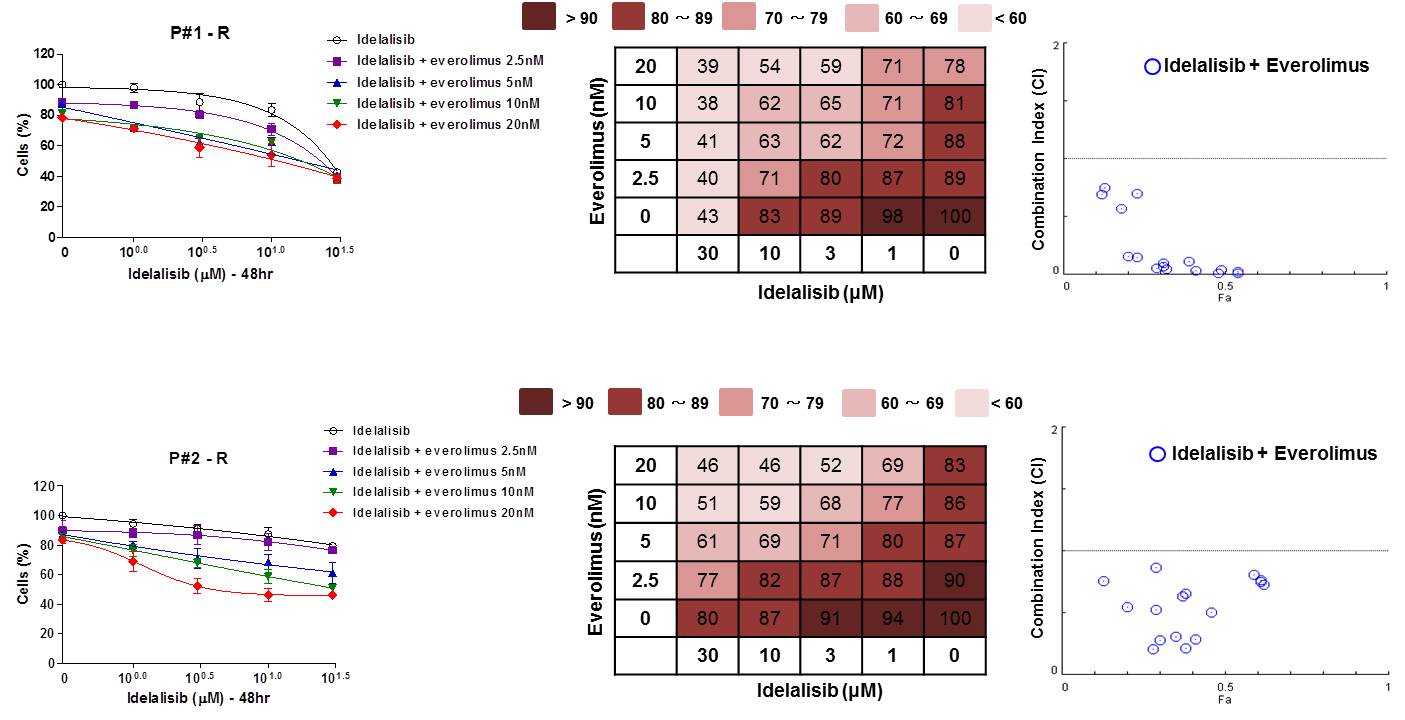
**

**Supplementary Figure 4.**

**
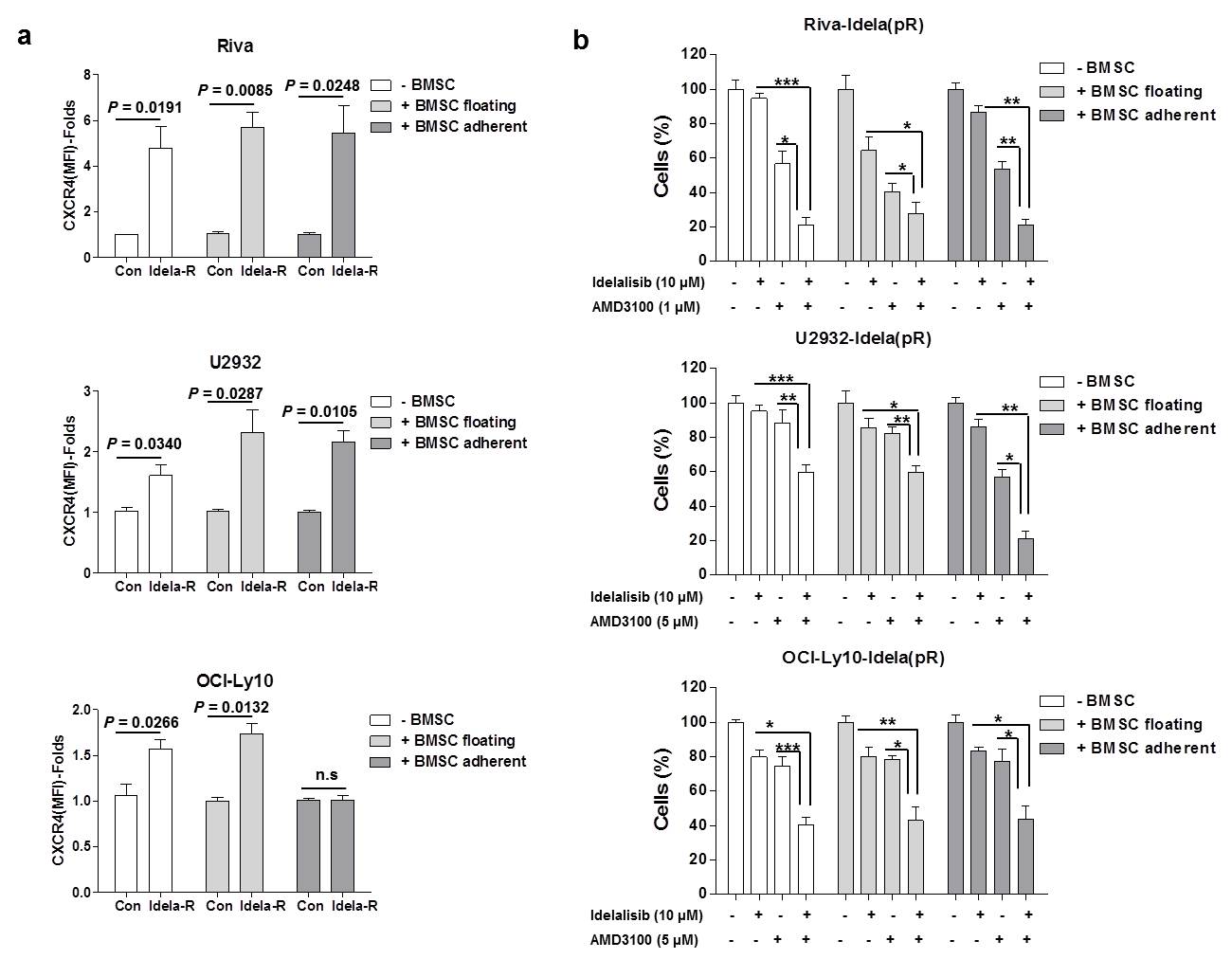
**

**Supplementary Figure 5.**


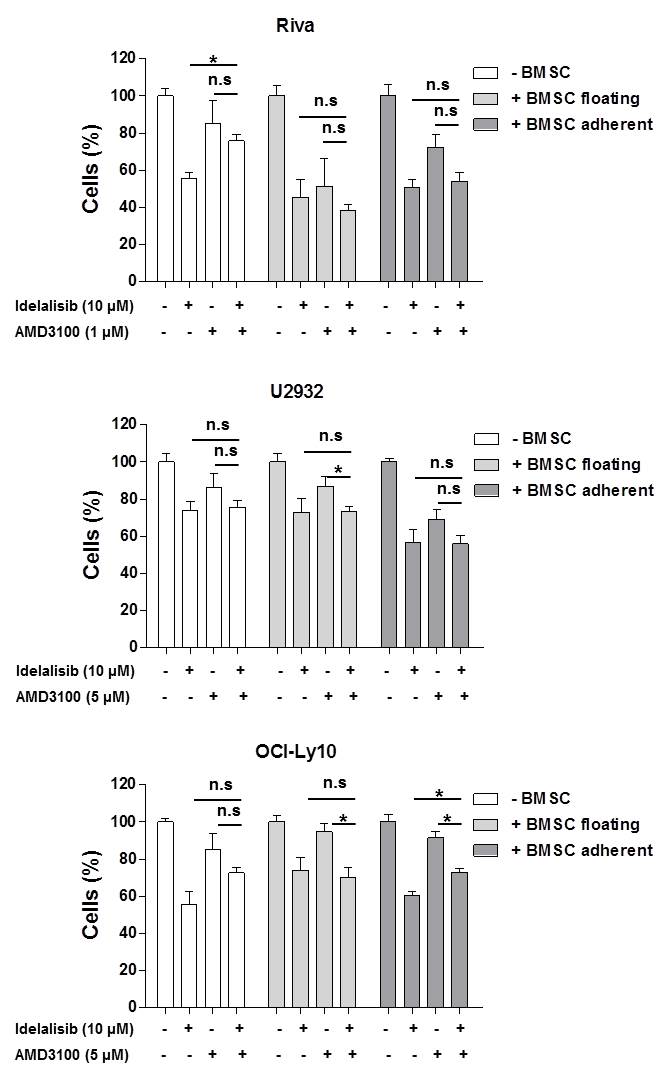


**Supplementary Figure 6.**

**
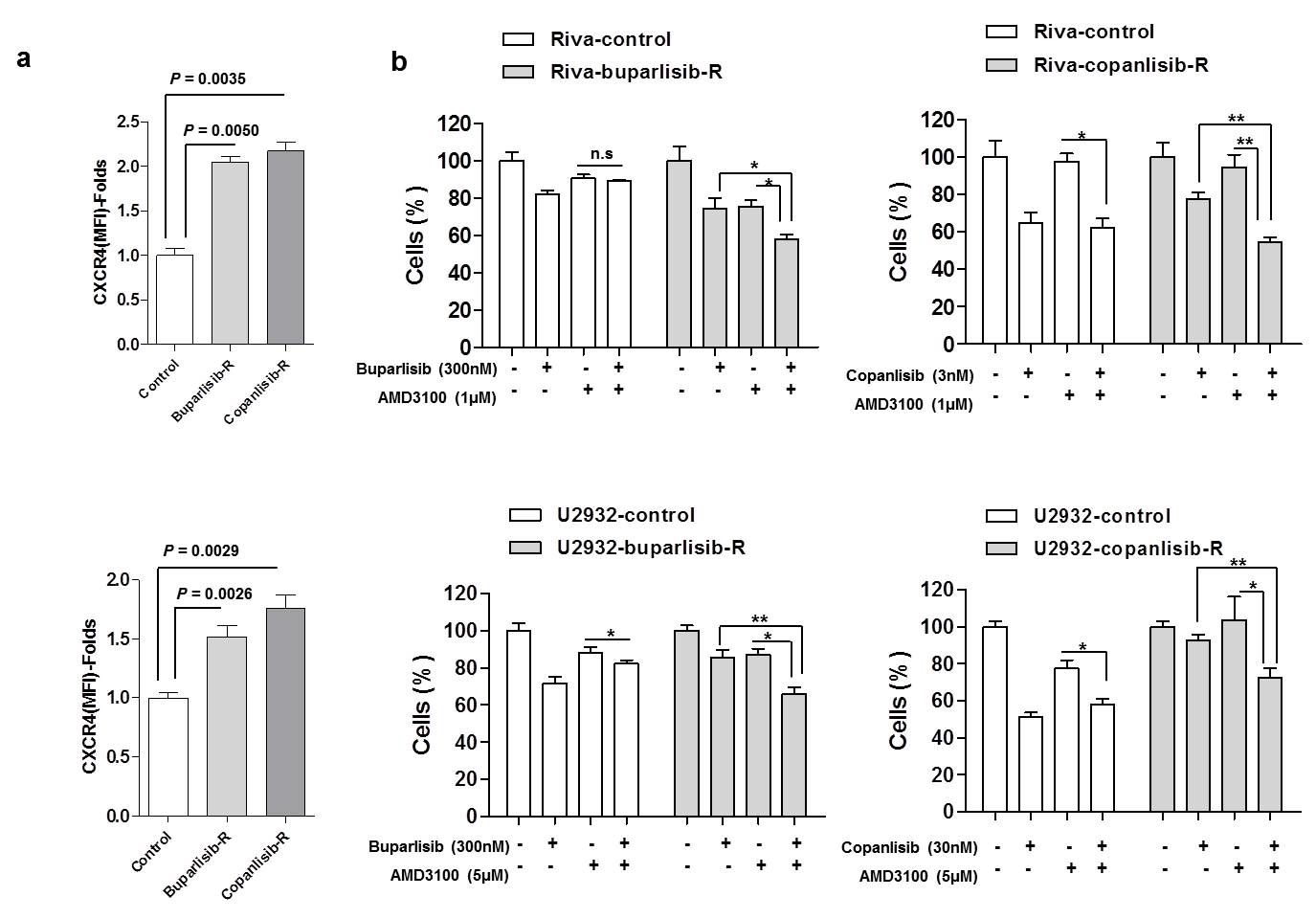
**

**Supplementary Figure 7.**

**
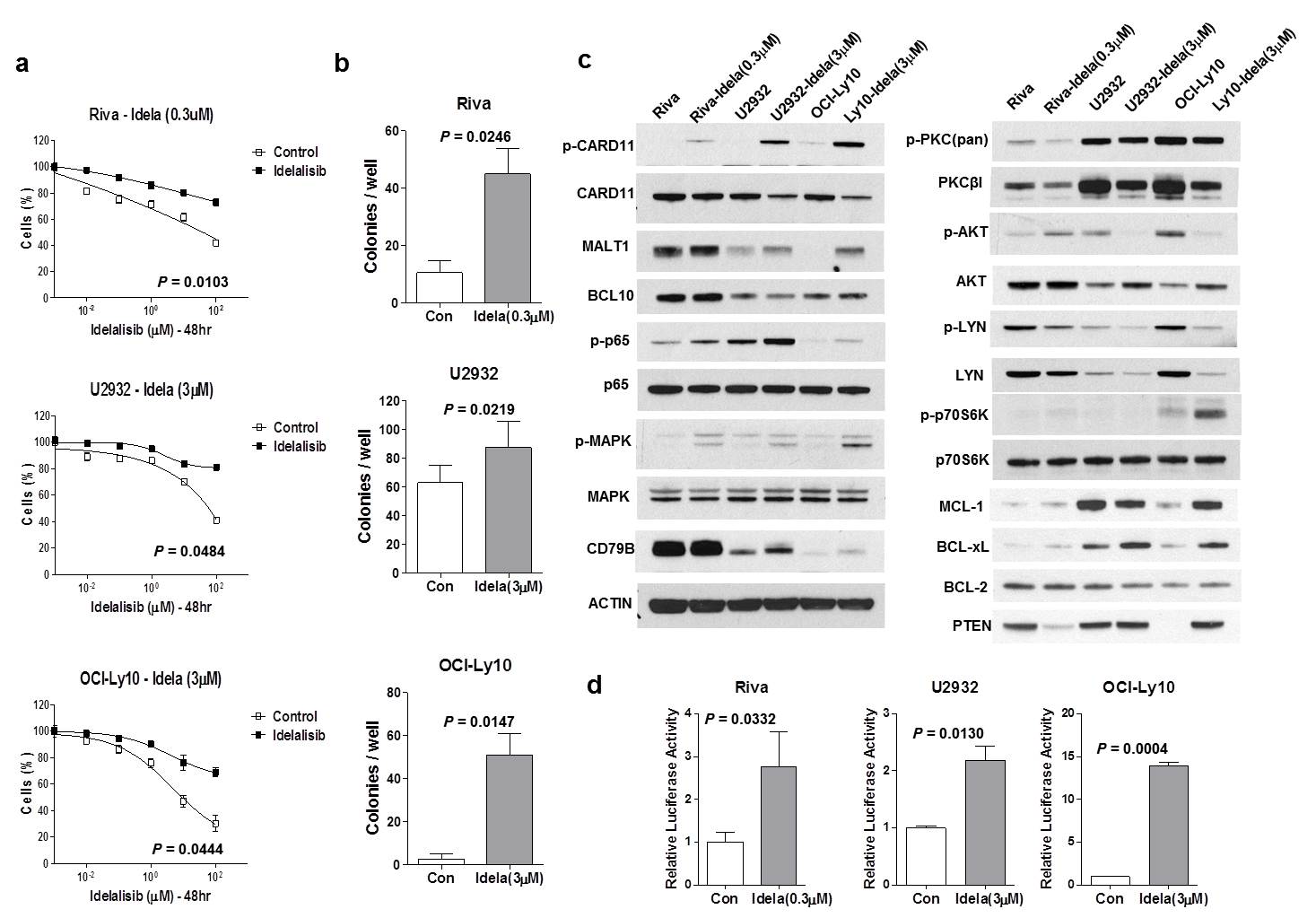
**

**Supplementary Figure 8.**

**
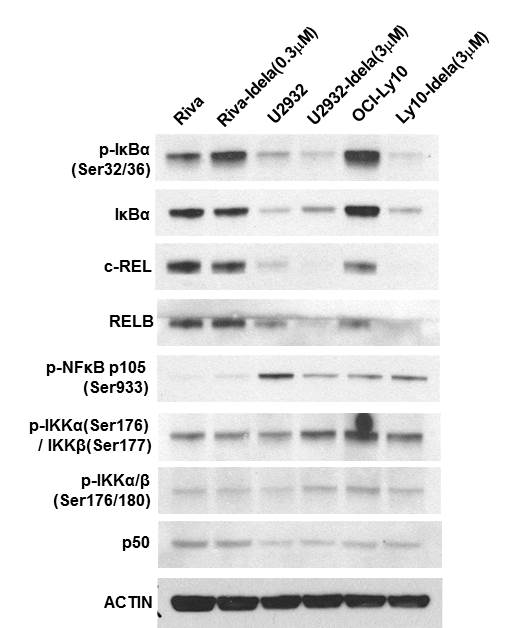
**

**Supplementary Figure 9.**

**
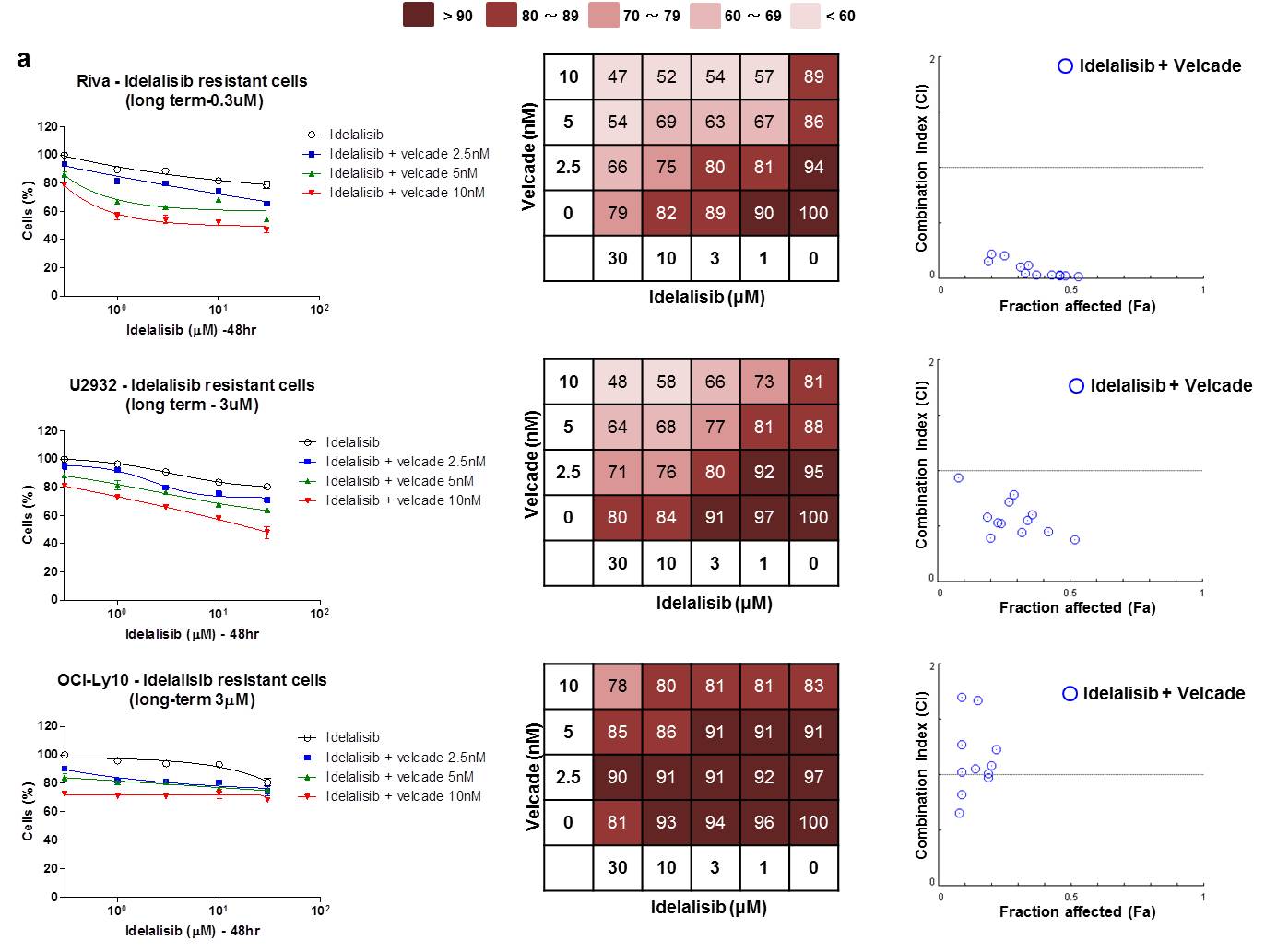
**

**
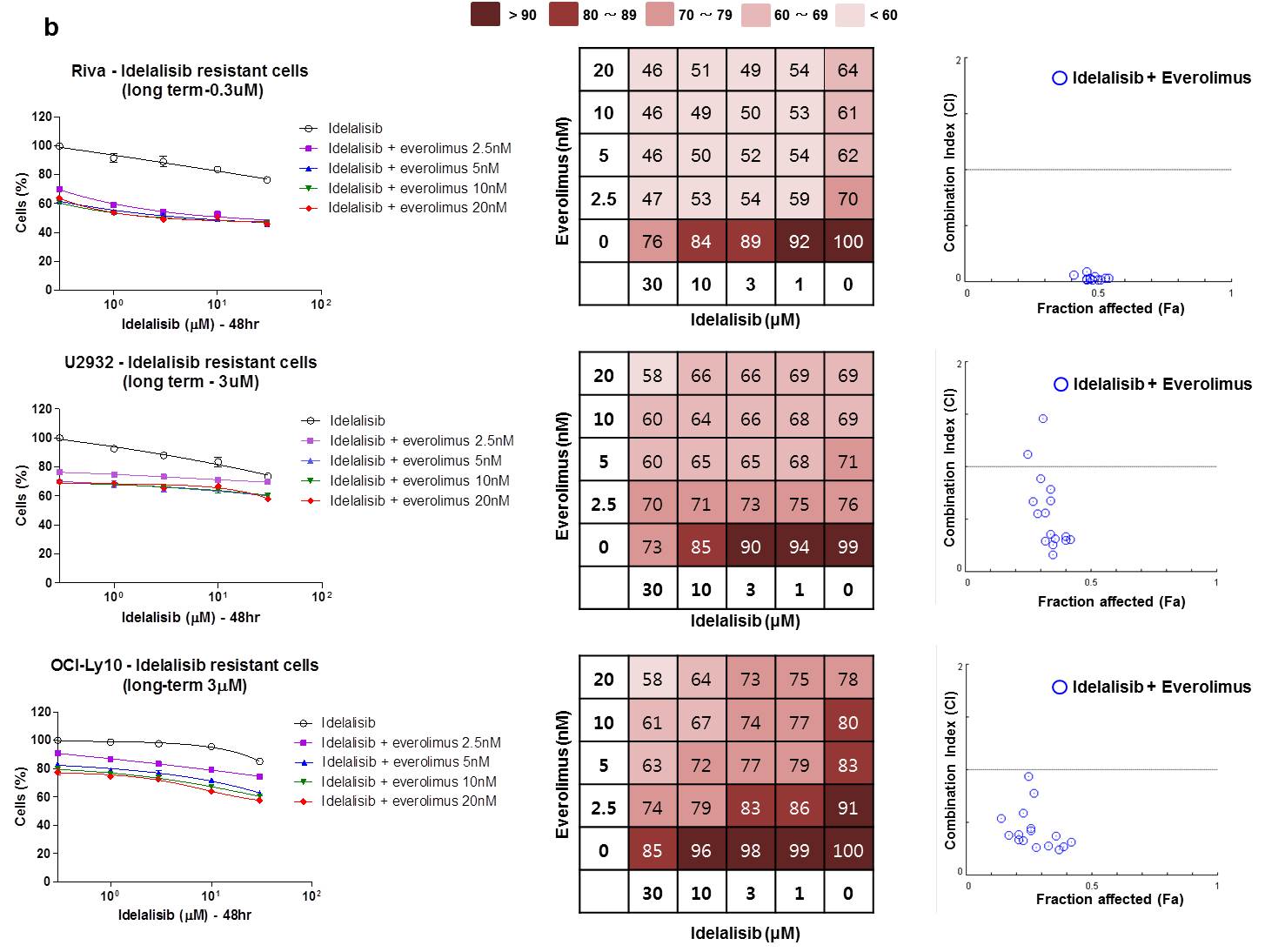
**

**Supplementary Figure 10.**

**
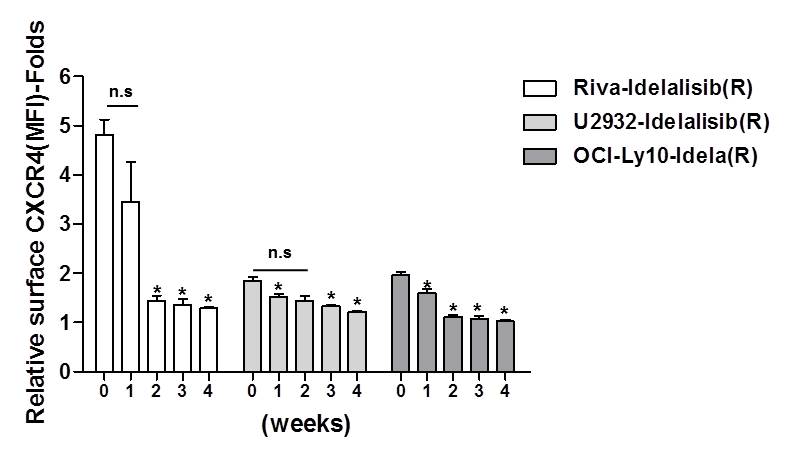
**
