# Supplementary material for: CXCR4 can induce PI3Kδ inhibitor resistance in ABC DLBCL
Source: Blood Cancer J. 2018 Feb 22;8(2):23. doi: 10.1038/s41408-018-0056-9 (PMC5823878; doi:10.1038/s41408-018-0056-9)
Supplement: Supplementary file 2 — Supplementary materials and method [file 41408_2018_56_MOESM2_ESM.docx]

**SUPPLEMENTARY MATERIALS AND METHODS**

**1. Cell lines, culture conditions, transfection and inhibitors**

Riva and U2932 cell lines were purchased from Leibniz-Institut DSMZ-Deutsche Sammlung von Mikroorganismen und Zellkulturen GmbH (Braunschweig, Germany). The HS-5 (bone marrow stroma) cell line was purchased from ATCC (Rockville, MD) and OCI-Ly10 obtained from Dr. Y. K. Jeon (Seoul National University Hospital, Seoul, Korea). Cell lines were cultured in RPMI 1640 (Riva and U2932), Dulbecco’s Modified Eagle’s medium (HS-5) or Iscove’s modified Dulbecco’s medium (OCI-Ly10) supplemented with heat-inactivated 10% (20% for OCI-Ly10) fetal bovine serum (FBS), penicillin and streptomycin (Gibco-BRL, Grand Island, NY) in a 5% CO_2_ -containing atmosphere. Primary refractory cells were isolated by collecting live cells after exposure of three parental ABC-DLBCL cell lines and primary human DLBCL cells (Riva, 20 μM; U2932, 30 μM; OCI-Ly10, 30 μM) to a lethal (IC90) dose of idelalisib for 72 h. Additionally, buparlisib (Riva, 1μM ; U2932, 3μM ) and copanlisib (Riva, 10nM; U2932, 1μM) were used for primary refractory cells. Live cells were collected with the FACSAria system (Becton Dickinson, CA, USA) and designated Riva-Idela(pR), U2932-Idela(pR), OCI-Ly10-Idela(pR) and pDLBCL(pR), respectively.

To generate cell lines with acquired idelalisib resistance, the respective parental cells were exposed to progressively increasing concentrations of idelalisib (termed Riva-Idela, U2932-Idela and OCI-Ly10-Idela) for 4 weeks. Idelalisib, AMD3100, Everolimus and Velcade were purchased from Selleck Chemicals (Houston, TX77054).

**2. Apoptosis assay**

An Annexin-V-fluorescein isothiocyanate (FITC) Apoptosis Detection Kit (BD Biosciences, San Joes, CA) was used to detect apoptosis via flow cytometry (BD FACS Verse). Parental and idelalisib-resistant cells were exposed to idelalisib harvested, and processed according to the manufacturer’s instructions. Caspase-3/7 enzymatic activity was measured using the Caspase-Glo 3/7 Assay kit (Promega, Medison, WI) according to the manufacturer’s instructions.

**3. cDNA microarray analysis**

Genes expressed in idelalisib-resistant and control ABC-DLBCL (Riva, U2932 and OCI-Ly10) cells were analyzed on an Illumina HumanHT-12 v4 Expression BeadChip (Illumina, Inc., San Diego, CA). Target preparation and microarray processing procedures were performed as described in the Illumina Expression BeadChip Analysis Manual. The pre-processing procedures for cell intensity files (CEL) and subsequent microarray analyses were performed using Illumina GenomeStudio v2011.1 (Gene Expression Module v1.9.0). Data were subjected to global scale normalization. Differentially expressed genes were selected based on fold change and Student’s *t* -test data (over two-fold and *P* <0.01, respectively), compared with the corresponding controls.

**4. Antibodies for Western blot**

The antibodies employed included those specific for p-NF-κB(S536), p-AKT(S473), AKT, p-PKC(pan), p-p44/42 MAPK(T202/Y204), p44/42 MAPK, p-p70S6K(Thr389), p70S6K, p-CARD11(S652), CARD11, p-PKC(pan), p-LYN(Y507), MCL-1, PTEN, p-IκBα(S32/36), IκBα, c-REL, RELB, p-NF-κB p105(S933), p-IKKα(S176)/IKKβ(S177), p-IKKα/β(S176/180), p50 (Cell Signaling, Beverly, MA), MALT1, BCL10, CD79B, PKC βI, LYN, NF-κB, BCL-xL, and BCL-2 (Santa Cruz, CA), with ACTIN (Sigma) as a loading control.

**5. Measurement of CXCR4 expression**

Harvested cells were washed in PBS and incubated at 4° C with APC-conjugated anti-CXCR4 (BD Pharmingen, BD Biosciences) or APC-conjugated isotype control (R&D Systems, MN 55413, USA) in 2% FBS-PBS for 1h, washed twice, and resuspended in PBS. Cells were analyzed via flow cytometry using FACS Verse.

**6. Cell migration assay**

The migration assay was conducted in triplicate using 5-μm pore size Transwells (Costar) as described.^11^ The bottom compartment was filled with 600 μL of 1% FBS containing (250 ng/mL) CXCL12 (PeproTech EC), and 5 x 10^5^ cells in 100 μL of 1% FBS medium applied to the top compartment. After 4 h of incubation, the amount of cells that migrated to the bottom compartment was determined via FACS Verse (BD Biosciences)..

**7. Assessment of cell viability**

Drug effects on cell viability were monitored using trypan blue staining. Alternatively, the CCK-8 viability assay was used. For the CCK-8 assay, cells were incubated in triplicate in a 96-well plate in the presence or absence of the indicated test samples in a final volume of 0.1 mL for 48 h at 37°C, followed by addition of 20 μL Cell Counting Kit-8 (CCK-8; Dojindo Laboratories, Kumamoto, Japan) to each well. After 2 h incubation at 37°C, optical density (OD) at 450 nm was measured using a 96-well multiscanner autoreader. Cell viability was expressed as a percentage (OD of the experimental sample/OD of control).

**8. Co-culture experiments**

HS-5 (BMSC) cells were plated at a density of 2 x 10^4^ cells per well in 24-well plates and incubated overnight. The next day, ABC DLBCL cells were seeded on top of HS-5 cells, either alone or in combination with AMD3100 and idelalisib, at a density of 2 x10^5^ cells/mL in medium supplemented with 1% FBS as described.^11^ After 48 h co-incubation, nonadherent cells were collected and the adherent cell fraction harvested with trypsin/EDTA. Cells were washed with PBS and analyzed via FACS for viability and CXCR4 expression. Cells were counterstained with anti-CD20, and ABC DLBCL cells distinguished from BMSCs after gating on CD20+ lymphoma cells.

**9. Soft agar colony formation**

The soft agar colony formation assay was performed by seeding cells (1 x 10^4^/ six-well plate) in a layer of 0.4% agar-RPMI-FBS over a bottom layer of 0.8% agar-RPMI-FBS. Cultures were maintained at 37°C. On day 14, cells were fixed with pure ethanol containing 0.05% crystal violet, and colony formation efficiencies quantified by counting. OCI-Ly10 cells were seeded in six-well plates (500/well) in triplicate in MethoCult H4100 (Stem Cell Technologies) for 14 days. After this time, colonies were counted and images acquired with the Gel Doc XR+ system (Bio-Rad).

**10. Luciferase assay**

The NF-κB reporter construct and pRL-TK vector were obtained from Promega. The reporter construct and *Renilla reniformis* luciferase-producing vector, pRL-TK were transfected into ABC DLBCL-idelalisib resistant cells using the Amaxa electroporation system. Firefly and Renilla luciferase activities were measured with the Dual-Luciferase Reporter Assay system, according to the manufacturer’s instructions (Promega).
